# COMING TOGETHER: HOW AFRICAN AMERICAN ADULT DAUGHTER ALZHEIMER’S CAREGIVERS ARE NAVIGATING FAMILY RELATIONSHIPS

**DOI:** 10.1093/geroni/igae098.2266

**Published:** 2024-12-31

**Authors:** Deneisha Scott-Poe, Megan Dolbin-MacNab

**Affiliations:** Converse University, Spartanburg, South Carolina, United States; Virginia Tech, Blacksburg, Virginia, United States

## Abstract

Alzheimer’s disease currently impacts around 5.8 million Americans, with African Americans being two to three times more likely to develop Alzheimer’s disease than their white counterparts. Most of these individuals are being cared for by their adult daughters. Previous research has explored African American women’s reports of less caregiver burden and strain, yet few studies have examined the impact of racial and gender oppression on how African American adult daughters approach caregiving. Guided by the theoretical perspective of intersectionality, the purpose of this qualitative study was to gain a deeper understanding of the family dynamics of 26 African American adult daughter caregivers and how the intersection of race, gender, and the history of care influences caregivers and their family relationships. To participate in the study, individuals had to at least be 18, be providing care for one year, and self-identify as African American and woman. Results of a constructivist grounded theory analysis revealed four major themes. The first was contextual stressors, which highlighted the unique sets of stressors that impacted caregiver mental health. The next theme was ideology about African American women and care, which emphasized the familial and personal beliefs about how women cared. The third theme, expectations placed on caregivers, highlighted the caregivers’ internalization of how women are supposed to care. The last theme, coming together, highlights how caregivers navigated their family relationships while providing care to their parents with Alzheimer’s disease. Implications for future research and systemic practice with African American adult daughter caregivers will be discussed.

